# The impact of improved water supply on cholera and diarrhoeal diseases in Uvira, Democratic Republic of the Congo: a protocol for a pragmatic stepped-wedge cluster randomised trial and economic evaluation

**DOI:** 10.1186/s13063-021-05249-x

**Published:** 2021-06-21

**Authors:** Karin Gallandat, Aurélie Jeandron, Ian Ross, Jaime Mufitini Saidi, Baron Bashige Rumedeka, Vercus Lumami Kapepula, Simon Cousens, Elizabeth Allen, Amy MacDougall, Oliver Cumming

**Affiliations:** 1 Department of Disease Control, London School of Hygiene and Tropical Medicine, London, UK; 2 Ministère de la Santé Publique, Division Provinciale de la Santé Publique, District Sanitaire d’Uvira, Uvira, South Kivu Democratic Republic of the Congo; 3 Department of Hydrology, Centre de Recherche en Hydrobiologie, Uvira, South Kivu Democratic Republic of the Congo; 4 Department of Infectious Disease Epidemiology, London School of Hygiene and Tropical Medicine, London, UK; 5 Department of Medical Statistics, London School of Hygiene and Tropical Medicine, London, UK

**Keywords:** Water supply, Infrastructure, WASH, Cholera, Diarrhoea, Stepped-wedge cluster randomised trial

## Abstract

**Introduction:**

Diarrhoeal disease remains a leading cause of mortality and morbidity worldwide. Cholera alone is estimated to cause 95,000 deaths per year, most of which occur in endemic settings with inadequate water access. Whilst a global strategy to eliminate cholera by 2030 calls for investment in improved drinking water services, there is limited rigorous evidence for the impact of improved water supply on endemic cholera transmission in low-income urban settings. Our protocol is designed to deliver a pragmatic health impact evaluation of a large-scale water supply intervention in Uvira (Democratic Republic of the Congo), a cholera transmission hotspot.

**Methods/design:**

A stepped-wedge cluster randomised trial (SW-CRT) was designed to evaluate the impact of a large-scale drinking water supply intervention on cholera incidence among the 280,000 inhabitants of Uvira. The city was divided into 16 clusters, where new community and household taps will be installed following a randomised sequence over a transition period of up to 8 weeks in each cluster. The primary trial outcomes are the monthly incidence of “confirmed” cholera cases (patients testing positive by rapid detection kit) and of “suspected” cholera cases (patients admitted to the cholera treatment centre). Concurrent process and economic evaluations will provide further information on the context, costs, and efficiency of the intervention.

**Discussion:**

In this protocol, we describe a pragmatic approach to conducting rigorous research to assess the impacts of a complex water supply intervention on severe diarrhoeal disease and cholera in an unstable, low-resource setting representative of cholera-affected areas. In particular, we discuss a series of pre-identified risks and linked mitigation strategies as well as the value of combining different data collection methods and preparation of multiple analysis scenarios to account for possible deviations from the protocol. The study described here has the potential to provide robust evidence to support more effective cholera control in challenging, high-burden settings.

**Trial registration:**

This trial is registered on clinicaltrials.gov (NCT02928341, 10th October 2016) and has received ethics approval from the London School of Hygiene and Tropical Medicine (8913, 10603) and from the Ethics Committee from the School of Public Health, University of Kinshasa, Democratic Republic of the Congo (ESP/CE/088/2015).

## Abstract (fr)

**Resume (version française):**

*Please note this translation has been generated by the authors and has not been checked against the original, peer-reviewed English version by the Journal. Any discrepancies between the two versions should be raised with the authors.*

*Cette traduction a été préparée par les auteurs et sa conformité avec la version anglaise revue par les pairs n’a pas été vérifiée par le journal. Toute différence entre les deux versions doit être signalée aux auteurs.*

**Introduction:**

Les maladies diarrhéiques restent une cause majeure de mortalité et morbidité dans le monde. Le choléra seul cause environ 95’000 morts par an, dont la plupart dans des contextes endémiques où l’accès à l’eau est inadéquat. Tandis qu’une stratégie globale pour l’élimination du choléra d’ici à 2030 appelle des investissements dans l’amélioration des services d’approvisionnement en eau, il y a peu de données probantes et rigoureuses sur l’impact d’un approvisionnement en eau amélioré sur la transmission endémique du choléra dans les contextes urbains à faibles ressources. Notre protocole d’étude est conçu pour livrer une évaluation d’impact épidémiologique pragmatique d’une intervention d’approvisionnement en eau à large échelle à Uvira, République Démocratique du Congo, un point focal de transmission du choléra.

**Méthodes/Conception:**

Un essai randomisé par grappes par échelons *(stepped-wedge cluster randomised trial, SW-CRT)* a été conçu pour évaluer l’impact d’une intervention d’approvisionnement en eau potable à large échelle sur l’incidence du choléra parmi les 280'000 habitants d’Uvira. La ville a été divisée en 16 grappes, dans lesquelles de nouvelles bornes fontaines et branchements individuels seront installés suivant une séquence randomisée sur une période allant jusqu’à 8 semaines par grappe. Le principal résultat de l’essai sera l’incidence mensuelle des cas de choléra « confirmés » (patients positifs par tests de détection rapide) et des cas « suspects » (patients admis au centre de traitement du choléra). Des évaluations de processus et économique menées en parallèle fourniront des informations complémentaires sur le contexte, les coûts et l’efficience de l’intervention.

**Discussion:**

Dans ce protocole, nous présentons une approche pragmatique pour effectuer une recherche rigoureuse visant à évaluer les impacts d’une intervention complexe d’approvisionnement en eau sur la diarrhée sévère et le choléra dans un contexte instable et à faibles ressources, représentatif des zones affectées par le choléra. En particulier, nous considérons une série de risques pré-identifiés et les stratégies de mitigation associées ainsi que la valeur de combiner différentes méthodes de collecte de données et de préparer de multiples scénarios d’analyse pour tenir compte d’éventuelles déviations du protocole. L’étude présentée ici a le potentiel de fournir des évidences scientifiques robustes pour soutenir des stratégies de contrôle du choléra plus efficientes dans les contextes difficiles qui sont fortement affectés.

**Enregistrement de l’essai:**

Cet essai est enregistré sur clinicaltrials.gov (NCT02928341, 10 octobre 2016) et a reçu les approbations éthiques de la London School of Hygiene and Tropical Medicine (8913, 10603) ainsi que de l’Ecole de Santé Publique de l’Université de Kinshasa, République Démocratique du Congo (ESP/CE/088/2015).

## Introduction

Despite substantial progress in recent decades, diarrhoeal disease remains a leading cause of mortality and morbidity worldwide [1, 2]. In 2015, an estimated 2.3 billion cases of diarrhoea occurred globally, including almost one billion cases in children under five, causing half a million deaths, mostly in sub-Saharan Africa and South Asia [1]. Early childhood diarrhoea may have long-term consequences including stunting and impaired cognitive development [3, 4]. A broad range of enteric pathogens can cause diarrhoeal disease, including Rotavirus, *Cryptosporidium* spp., *Shigella* spp., and enterotoxigenic *E. coli* (ETEC), with aetiology varying by age and setting [1, 5].

The diarrhoeal disease cholera is caused by toxigenic *Vibrio cholerae* O1 or O139 and is characterised by acute watery diarrhoea. Cholera remains an important cause of diarrhoeal disease morbidity and mortality, with an estimated 1.3 to 4 million cases and 95,000 deaths globally in 2015 [6]. Approximately half of cholera deaths occur in children under five [7], and the cholera burden is concentrated in endemic settings in Asia and Africa [6]. In recent years, large outbreaks in Haiti [8] and in Yemen [9] have highlighted the vulnerability to cholera of populations affected by humanitarian crises [10–13]. In sub-Saharan Africa, identified transmission “hotspots” which represent less than 4% of the region’s total population account for over half of the total cholera morbidity burden [14].

In response to the ongoing challenge of cholera prevention and control, the World Health Organization (WHO)-led Global Task Force on Cholera Control (GTFCC) has set an international goal of reducing cholera deaths by 90% by 2030 [15]. In order to achieve this ambitious target, the GTFCC promotes a multisectoral approach targeting transmission hotspots via three axes [15]: (a) surveillance and health systems strengthening; (b) vaccination campaigns; and (c) long-term improvements in water, sanitation, and hygiene (WASH) services.

The first axis of surveillance and monitoring of cholera is often hindered by weak national health systems in those countries where cholera is endemic [6, 16–18], and there is substantial underreporting [6]. Another challenge lies in the broad criteria of the WHO’s cholera case definition [19] which defines a “suspected cholera case” in endemic areas as presentation of acute watery diarrhoea with or without vomiting. These broad clinical criteria can lead to misdiagnosis of cholera cases, and whilst WHO recommends laboratory confirmation, suspected cases are not consistently confirmed by laboratory methods [18]. Studies from several African countries [20–22] and from Haiti [23, 24] found laboratory confirmation by culture of between 0 and 63% of suspected cholera cases.

The second axis, vaccination campaigns, is supported by oral cholera vaccines (OCV) which can provide protection against cholera for 2 to 4 years [25, 26] and indirect protection via herd immunity has also been documented [27–30]. A global stockpile was created in 2013 to ensure OCV availability for cholera control in outbreaks and humanitarian emergencies [31]. As OCV supply capacity increased in recent years, mass vaccination campaigns in transmission hotspots, in conjunction with improvements in WASH services, are now the foundation of the GTFCC strategy to eliminate cholera along with effective surveillance [15].

The third axis is long-term improvements in WASH services for cholera prevention, which aligns with Sustainable Development Goal (SDG) 6: universal access to safely managed water and sanitation services by 2030 [32]. Diarrhoeal diseases such as cholera are generally transmitted faecal-orally via environmental routes such as contaminated water or food [33]. WASH interventions can interrupt this environmental transmission and substantially reduce diarrhoeal disease risk [34]. However, 785 million people still did not have access to basic drinking water services in 2017 [35] and in a recent analysis, 60% of the diarrhoeal disease global morbidity burden was attributable to inadequate WASH services [36]. Improving the microbial quality of drinking water can prevent waterborne (and foodborne, where water is used in food preparation) transmission of diarrhoeal diseases whilst improving the supply or quantity of water available can prevent water-washed transmission of diarrhoeal diseases through enabling better personal and domestic hygiene. The latter may be key to cholera control, as recent studies have highlighted the importance of cholera transmission within households during outbreaks [37–41].

Whilst the link between drinking water access and risk of diarrhoeal disease and cholera is well established, there is limited evidence in the scientific literature on the impacts of large-scale improvements in water supply or sanitation infrastructure intended to reduce the burden of diarrhoeal diseases and cholera [34, 42]. This research gap is likely due to methodological as well as logistical and financial challenges related to conducting rigorous evaluations of technically complex, long-term interventions. Research challenges are enhanced in the complex, resource-limited, and often unstable contexts where the cholera burden is concentrated [13, 43] and the health impacts of WASH interventions are under-researched in humanitarian settings [44].

Since 2012, the Agence Française de Développement (AFD) and the Veolia Foundation (VF), with support from the European Union, have invested approximately 13 million euros in improvements to the water supply infrastructure in Uvira (Democratic Republic of the Congo, DRC), an identified hotspot for cholera transmission [14]. They commissioned an independent evaluation of the health impact of the planned works. This project offered a rare opportunity to investigate how improvements in urban water supply infrastructure, in a conflict-affected, cholera-endemic area, influence the incidence of cholera and other diarrhoeal diseases. In light of the potential public health value of such research but with consideration of the multiple challenges for implementation in this setting, we employed a deliberately pragmatic approach to this study protocol. The aim of this study is to evaluate the impacts of water supply infrastructure improvements on cholera and other diarrhoeal diseases in Uvira (DRC). We have identified a number of potential risks to the study and then pre-specified mitigative measures that would permit the assessment of health impact under different scenarios. This protocol and analysis plan are shared here in accordance with best practice principle for pre-specification but also to stimulate debate in the global health sector on strategies for undertaking rigorous research in unstable settings.

## Methods—trial design

Our study combines an open-cohort, stepped-wedge, cluster randomised trial (SW-CRT) and interrupted time series (ITS) analysis, with cross-sectional surveys of water-related practices, an economic and a process evaluation. Our approach is deliberately pragmatic with the different study components designed to mitigate a range of pre-identified risks to this study which relate to the delivery of a complex intervention in a challenging context.

Methods are described in accordance with the SPIRIT guidance [45] and the Consolidated Standards of Reporting Trials (CONSORT) extension for SW-CRT [46] to ensure adequate inclusion and reporting of key design and analysis components during development of this protocol. The SPIRIT check-list is available in Additional file 1.

### Aim and objectives

The aim of this study is to evaluate the impact of improved water services on cholera and other diarrhoeal diseases in the town of Uvira (DRC). The primary objective is to assess changes in the monthly incidence of suspected and confirmed cholera cases admitted to the main cholera treatment centre (CTC) in Uvira (using SW-CRT and ITS analyses). Additional objectives are to assess (1) changes in domestic water-related practices (using cross-sectional surveys, and REGIDESO operational and billing information); (2) cost-effectiveness and cost-benefit of water supply improvements (through an economic evaluation); and (3) fidelity and compliance of the planned water supply intervention (through a process evaluation). Each of these study components is described below, followed by the analysis plan, household surveys, and economic and process evaluations.

### Study setting

Uvira is a town of approximately 280,000 inhabitants on the shore of Lake Tanganyika, in Eastern DRC (South Kivu) [47]. The region is characterised by protracted conflict and ethnic violence [48], with at least 100 active armed groups identified in South and North Kivu as of 2019 [49] and continuing, massive population displacements [50].

Cholera has been endemic in the region since the 1970s and Uvira is located within an internationally designated transmission “hotspot” [14, 51]. The CTC located in the General Hospital in Uvira is the main care structure for acute diarrhoea cases—defined as three or more loose or watery stool per day—for the city and provides free treatment including rehydration and, in some cases, antibiotics [21]. CTC admissions have been recorded by the study team since 2009, with an average of 1266 patients admitted annually or 3.4 admissions per year per 1000 inhabitants.

The WHO/UNICEF Joint Monitoring Program estimates that in 2017 approximately 36% of households in DRC relied on unimproved drinking water services and an additional 9% used surface water as their primary source [35]. The existing water supply infrastructure in Uvira was established in the colonial era (1958) [52] and was severely damaged during the First Congo War (1996–1997), with parts then repaired and/or modified by NGOs in the early 2000s. In 2018, before the planned intervention began, the water supply system in Uvira provided a partial and intermittent piped water service that reached an estimated 31% of the population [52]. Surveys conducted by the study team in preparation for the trial in 2016 and 2017 indicated that almost half (47%) of the participants (747 respondents from 458 households) reportedly used surface water—lake (13%) or rivers (34%), about a third (34%) used a tap outside their compound, and 18% had a tap in their compound as primary drinking water source (unpublished data).

### Rationale for using a cluster design and rationale for using a stepped-wedge design

A SW-CRT design was selected due to the nature and scale of the intervention, which involves the rehabilitation of structural water supply network components as well as the extension of that network. A cluster randomised design was selected because infrastructure improvements (e.g. piped network rehabilitation and extension or installation of community taps) will affect large numbers of water service users simultaneously. The stepped-wedge design overcomes the logistical constraint of not being able to deliver the intervention concurrently to all clusters due to the duration of the construction works. It also addresses ethical concerns by allowing water supply improvements to be delivered to the entire town of Uvira without causing delays. SW-CRT designs have been used previously for the evaluation of large-scale water treatment, supply, and/or management interventions in India [53], Bangladesh [54], and Mexico [55] but never for systems which include new household and community water connections, and never in a cholera-endemic setting.

### Intervention

The water supply intervention consists of a package of infrastructure works designed to improve the piped water service in Uvira (Fig. 3).

First, core components of the water supply network will be targeted and these works are expected to affect all clusters. The water treatment plant will be retrofitted with a system including flocculation, settling, sand filtration, and chlorination, and new pumps will be installed to double the water treatment plant production capacity. A new tank (2000 m^3^) will be built, increasing the available hydraulic head for water distribution and doubling the overall storage capacity for the town. Structural pipes (diameter ≥ 250 mm) will be installed and those with identified deficiencies will be rehabilitated (7.4 km total).

Second, at the cluster level, the secondary network will be extended to increase coverage and create loops, which are expected to improve the reliability of water distribution locally. The installation of 116 (expected range 100–120) new community taps is planned within the project, along with works on 3000 household connections, including approximately 1000 new taps for households that are currently not connected to the water supply network. While the total number of household connections (new and rehabilitated) included in the project is set to 3000, the total number of new household connections and the distribution of these connections between clusters will remain flexible for the reasons described in more detail below. Household-level connections and community taps will be installed in each cluster following the randomised allocation sequence (Table 1).

The Uvira water network is, and will continue to be, managed by the national company Régie de Distribution d’Eau de la République Démocratique du Congo (REGIDESO). Three committees will be formed by community members for the management of community taps with the support of a Congolese NGO that has led successful community engagement projects (ADIR). These committees will be responsible for the daily management of water distribution at the community taps and the collection of water consumption fees from users to pay monthly bills sent by REGIDESO to each committee.

REGIDESO will be responsible for the promotion of new household connections in each neighbourhood/cluster via appropriate communication channels. New household connections will be installed on a “first come, first served” basis in each cluster after interested individuals submit an application form to REGIDESO and pay a fee, partially subsidised by the project. The number of new household connections planned in each cluster is based on three parameters as part of the works planning (Table 1): the number of connections per 1000 inhabitants in each cluster, the proportion of the population with access to a tap within 300 m, and the network capacity in terms of number of total expected connections per meter of pipe within the cluster. However, given the reliance on individual demand for the installation of new household connections, and potential variability thereof, the team supervising construction works will be given discretion to reallocate new household connections to different clusters. Alternatively, in an attempt to facilitate the roll-out of the intervention, they may increase the proportion of household connections to rehabilitate according to identified needs and demands.

The management structure for the construction works is as follows: VF is leading the supervision of the works, including adhesion to the study protocol, with regular visits to the construction site and has contracted an engineering consulting firm for daily follow-up activities. The construction company was hired by REGIDESO, with AFD and VF support, after an international call for tender and is bound by a contract that specifically requires works to be implemented following the cluster randomisation protocol for the SW-CRT described below.

### Description and diagram of trial design

**Fig. 1:**
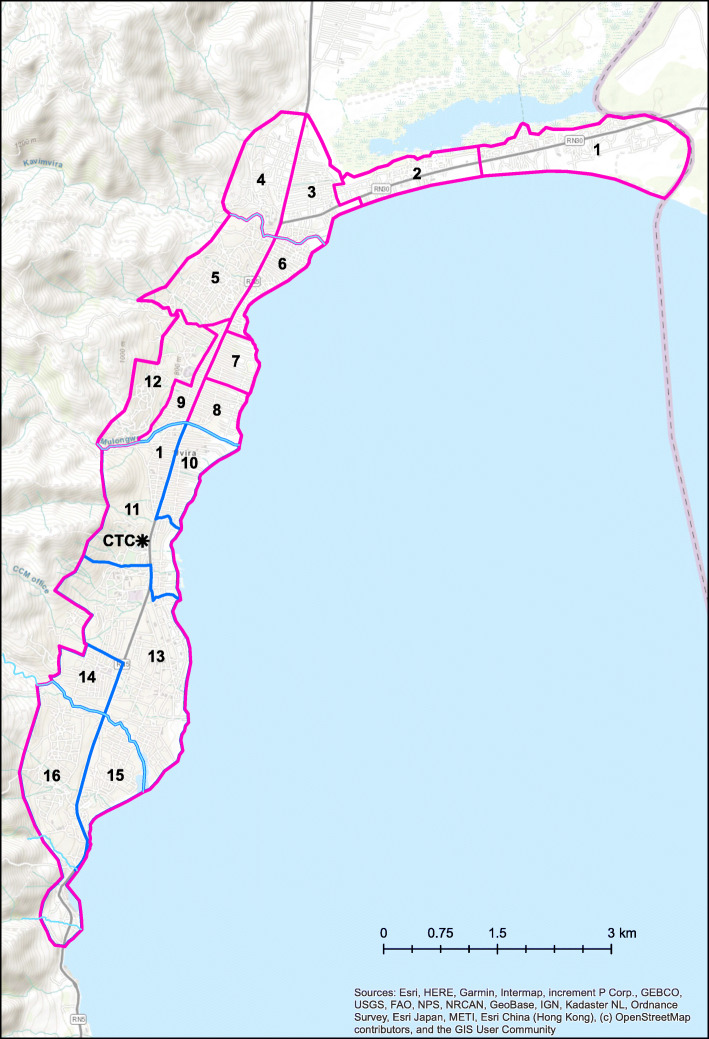
Map of Uvira with the definition of clusters and groups: South (blue, *n* = 6) and North (pink, *n* = 10)

The town of Uvira was divided into sixteen clusters stratified into two groups due to the project funding structure and planned works sequence: South (*n* = 6, areas primarily served by the existing tank) and North (*n* = 10, areas primarily served by the new tank) (Fig. 1). The existing and new tank will both eventually belong to the same water supply network and contribute to improving water service across all clusters. The primary criteria used to define clusters were the total length of pipes to be rehabilitated or installed and the number of new community taps; the secondary criteria included considerations such as alignment with existing administrative zones and natural features such as rivers. Key characteristics of the study clusters are presented in Table 1.

**Table 1:** Key cluster characteristics

Cluster #	Estimated population (March 2018)	Area [km^2^]	*Mean # suspected cholera cases per month	*Mean monthly incidence per 1000	Existing pipe length [m]	Pipe length to rehabilitate [m]	Length of new pipes to install [m]	# existing household connections	# provisionally planned new household connections	Number of planned new community taps	Allocation sequence
1	4291	2.02	1.9	0.4	2152	57	2628	16	66	6	15
2	4990	0.76	6.0	1.2	1578	1658	1792	89	25	3	16
3	11,183	0.87	6.4	0.6	5574	1266	1864	171	25	7	10
4	7328	1.02	5.4	0.7	2347	0	3398	25	100	5	12
5	13,649	1.27	4.4	0.3	2786	797	4177	19	205	4	9
6	8389	0.48	5.8	0.7	1730	499	2328	43	82	6	13
7	7480	0.37	2.0	0.3	4525	367	1065	152	26	2	8
8	15,455	0.43	4.8	0.3	2961	1097	1793	199	25	5	7
9	3994	0.36	9.4	2.4	3848	189	2446	183	25	8	11
10	14,175	0.59	7.1	0.5	8901	692	1520	525	25	7	1
11	56,106	1.70	14.5	0.3	8775	1599	2401	633	254	16	5
12	12,462	0.99	3.0	0.2	2292	1455	1844	22	193	1	14
13	50,764	2.75	10.4	0.2	17,781	1666	1266	1384	24	11	4
14	16,175	0.55	6.7	0.4	907	0	2549	35	220	5	3
15	15,183	0.81	7.2	0.4	4091	135	2420	161	62	9	2
16	19,338	1.98	10.6	0.6	1788	2061	2874	28	318	20	6
**Mean**	**16,310**	1.06	6.6	0.4**	72,033	13,538	36,363	3685	1675	7.2	–
Minimum	3994	0.36	1.9	0.2	907	0	1065	16	25	1	–
Maximum	56,106	2.75	14.5	2.4	17,781	2061	4177	1384	318	20	–

*Based on CTC admissions between January 1, 2009, and March 31, 2018, and population in March 2018

**Mean weighted by cluster population size

**Fig. 2:**
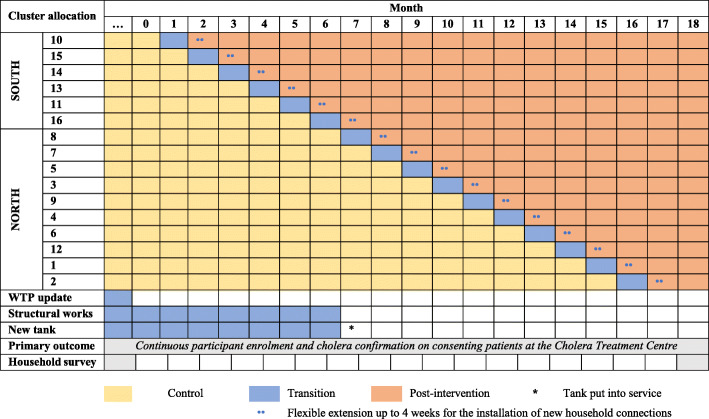
Trial diagram, inspired by [46]

Within each group (South and North), clusters were randomised to different initiation periods by the research team using a random number generator, with the intervention delivered to the South clusters first. The intervention interval—or “step”—during which time a given cluster receives the intervention was planned to be 4 weeks; this interval was extended to up to 8 weeks for the installation of new household connections to the water supply network (Fig. 2)—the reasons are discussed below.

### Participants

The population of interest is all inhabitants of Uvira, assuming that all Uvira inhabitants affected by severe diarrhoeal disease or cholera will eventually seek care or be referred to the CTC. All patients admitted to the CTC with suspected cholera are eligible for participation in the study and collection of a rectal swab for cholera confirmation (Fig. 2). No restriction is placed on patient age.

Following approval by the Ministry of Health for DRC, the CTC Coordinator can extract and share anonymised patient information as recorded in the CTC registry, including age, sex, address, dates and length of stay, and antibiotic treatment if any. All information that is extracted and included in this study protocol is anonymised with no personal identifiers included. Aggregated census data provided by municipal authorities will be used to estimate the population size for each cluster.

### Outcomes

Two primary outcomes will be used to assess the effectiveness of the intervention: (1) the monthly incidence of suspected cholera cases, as measured by the number of CTC admissions attributed to each cluster; (2) the monthly incidence of confirmed cholera cases, based on rapid detection test (RDT) results for eligible, consenting patients.

### Trial data collection

Information will be collected to characterise the water supply intervention as well as to monitor health outcomes.

Monitoring tools were developed to record progress of construction works on the water supply network. For each new community tap and each household connection in this project, a form will be completed that includes information on location (address, cluster number, GPS coordinates), owner (for household connections), type of connection (new or rehabilitated), and date of works completion. For household connections, the date of works completion will correspond to the date of actual service availability for the users. For community taps, the date of service availability may be different from that of works completion and will be recorded by management committee agents in the registers used to monitor water use.

Additionally, in order to further assess the intensity of the intervention, we will use the total volume of water distributed (m^3^/day) as measured at the exit of the pumping station and the total volume of water billed to users (m^3^/month per cluster), depending on data availability.

In parallel to the intervention, data will be collected at the CTC on an continuous basis, with all admissions and corresponding patient addresses recorded to allow assignment to study clusters, and rectal swabs collected from eligible, consenting patients for cholera confirmation via Crystal VC RDTs (Span Diagnostics, Surat, India) on alkaline peptone water enrichments, as described elsewhere [21]. The sensitivity and specificity of RDTs on enriched rectal swabs was estimated to be 92% and 91%, respectively [56]. Enriched samples testing positive via RDTs may be preserved on filter paper for *Vibrio cholerae* isolation and characterisation at a partner laboratory.

### Blinding

Blinding of participants to the intervention is not possible due to the nature of the intervention: hydraulic construction works are visible and affect water services where they are implemented. However, whether an individual lives in a control or intervention cluster at a given point in time is unlikely to affect care-seeking behaviour for acute diarrhoea and cholera confirmation remains an objective outcome, independent of a patient’s cluster status.

### Analysis plan

The SW-CRT and ITS analyses are detailed hereafter.

A generalised estimating equations (GEE) Poisson regression for rates will be used with robust standard errors. An indicator of water service quality will be included, which may integrate users’ distance to taps, hours of water availability, or volumes distributed, for example, to reflect improvements brought by the intervention in terms of water service. To account for seasonality a harmonic term for time (in months) will be included. This analysis accounts for correlations between individuals within clusters, as well as repeated measures of the same clusters over time. A small sample correction will be considered to adjust for the small number of clusters [57]. Note that data collected during the transition period for each cluster will not contribute to the analysis.

Since the intervention takes place over less than 2 years, the seasonal pattern of cholera infection may not be estimated correctly using only data from the trial period. A sensitivity analysis will be carried out using historical data from the cholera treatment centre which is available from 2009 to the start of the trial. The model will be re-fitted using the same covariates, with all historical data considered non-intervention time. We will also consider the inclusion of a treatment by time interaction term in the GEE model as part of further sensitivity analyses linked to seasonality.

An intention-to-treat analysis will be carried out for the main analysis. The above models will be repeated with the other primary outcome: rate of confirmed cholera cases per 1000 residents.

Randomisation may be particularly challenging to ensure given the nature of the intervention. If the randomisation scheme is not adhered to, balance between arms cannot be assumed. In this case, relevant confounders which may be associated with intervention allocation and outcome will be included in the main analysis, as they would be in non-randomised settings. These may include patient’s age and sex, and factors related to their household location such as water service quality and distance from the cholera treatment centre.

As a complement to the main stepped-wedge analysis, an additional analysis will be carried out which makes fewer assumptions about the structure of the intervention. An interrupted time series analysis will be fitted using historical data, with a fixed starting point for the intervention rather than staggered starting points as assumed in the analysis above. Data collected throughout the intervention until completion of the works in the last cluster will be excluded from the analysis. We intend to continue data collection for at least 12 months after completion of the intervention to allow comparison of suspected and confirmed cholera incidence before and after water service improvements.

### Sample size

The “steppedwedge” command was used within Stata 16 (StataCorp LLC, College Station, TX, USA) to calculate the minimum detectable difference (MDD) in the main SW-CRT analysis given known parameters. The pre-intervention cholera rate across all clusters was assumed to be 0.4 per 1000 people per month (Table 1). Power was set at 80% with a 5% significance level. The calculation was carried out with the intraclass correlation coefficient (ICC) set at 0.3 and mean cluster size 16,000. ICC, average cluster size and cholera rates were found using the historical data (Table 1). The MDD was 0.1 per 1000, corresponding to a decrease to 0.3 per 1000 people (or increase to 0.5 per 1000) or a 25% reduction in suspected cholera incidence. In a meta-analysis, availability of piped water on premises compared to unimproved water sources was found to reduce the risk of diarrhoeal disease in children by 23% (confidence interval 7–36%) [34].

A sample size calculation for the ITS analysis was also carried out. Follow-up periods of 6 and 12 months from an end date of July 2022 were considered. Mean cholera rates per month were estimated using historical data from the CTC in a quasi-Poisson model with a linear term for year and harmonic terms for month. These were used along with estimates of variance and over dispersion parameters to find the minimum detectable difference. The mean monthly rate per 1000 people was estimated to be 0.25 over a 12 month follow-up, 0.29 over 6 months. The minimum detectable reduction in cholera rates was therefore estimated to be 65% with a follow-up time of 12 months, and 75% for 6 months.

### Cross-sectional household surveys

During the inception phase of this study, a household survey was conducted in 2016 and repeated in 2017 (unpublished) to assess household water collection, treatment, storage, and use practices as well as drinking water quality before the intervention. An additional survey using similar questionnaires will be carried out upon completion of the intervention (Fig. 2) to assess whether changes in water service modified households’ water-related habits, including the quantity and source of water used for different purposes, water treatment and storage, and personal and household hygiene practices. Drinking water samples will also be collected from participating households to test for bacterial indicators of water quality (*E. coli* and total coliforms). Five hundred households will be randomly pre-selected across the 16 trial clusters and areas with different levels of access to piped water service post-intervention, using a similar sampling strategy as for the two previous surveys [58].

### Economic evaluation

The economic performance of the intervention will be assessed using cost-effectiveness analysis (CEA) and cost-benefit analysis (CBA). Both will use decision analytic modelling to compare costs and outcomes under the intervention scenario as compared to a “do nothing” scenario, over a 20-year time horizon. The CEA will assess the incremental cost per (i) confirmed case of cholera averted, (ii) CTC admission averted, and (iii) disability-adjusted life year (DALY) averted. These measures exclude the value of the broader outcomes of the intervention beyond infectious disease. Therefore, we will also undertake a CBA that will value health and other outcomes (such as time savings) in monetary terms and calculate a benefit-cost ratio. Cost data for these analyses will come from intervention financial records (capital costs), REGIDESO financial records and interviews (operational costs such as electricity, chemicals and staff), and household surveys (cost-of-illness, travel time to off-plot sources). Outcomes will be estimated from trial records.

### Process evaluation

**Fig. 3:**
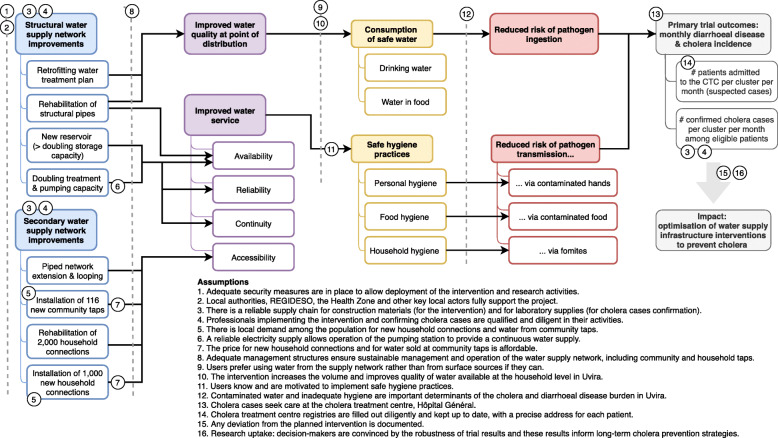
Conceptual framework for the Uvira trial

Assumptions on the pathway from intervention to impact (Fig. 3) will be assessed, if possible, by conducting a process evaluation alongside the trial. Information will be gathered from multiple sources: operational data from the water treatment and pumping stations, as well as billing information and community taps registries, in conjunction with household survey data, will allow us to document water service access and quality in each cluster throughout the project—thus helping understand fidelity and compliance of the planned intervention.

### Recruitment and consent

Recruitment of participants for cholera confirmation will occur continuously at the Uvira CTC, where trained study staff have offered participation to all admitted patients since April 2016. Written informed consent (Additional file 2) will be sought from all participants prior to collection of a rectal swab by trained CTC study staff. For patients under 15 years old, parental consent will be sought for participation in the study.

Recruitment of participants for household surveys will be done by trained enumerators following the random sampling scheme described above. Written informed consent will be obtained from an adult individual (over 18 years old) accepting to participate in the survey on behalf of each household.

### Data management

Anonymised data from the CTC registries will be transferred onto electronic tablets by trained study staff and sent onto a secure, encrypted online server via Open Data Kit (ODK) managed by the London School of Hygiene and Tropical Medicine (LSHTM), with daily back-ups. Regular data accuracy checks will be performed by research team members both online (weekly) and during visits to the study site.

Household survey data will also be collected electronically by trained enumerators using ODK, with encryption, and uploaded onto the same LSHTM-managed server. Daily data quality checks will be performed by research team members during household survey data collection periods so that any issues are addressed in a timely manner.

Authorised research team members will download data onto password-protected computers for analysis. Any identifying information will be stored separately from clinical records.

### Data monitoring

While regular ODK data quality checks will be performed by research team members, no data monitoring board was set up for this trial in view of the nature of the intervention (water supply improvements) and low risk to participants; for the same reason, no interim analyses are planned and stopping guidelines have not been established. Reporting adverse events will be the responsibility of the Principal Investigator.

### Auditing and governance

External audits of the trial are currently not planned. A steering committee was set up with representatives of all study partners, including the Ministry of Health, REGIDESO, funding agencies, and cholera response organisations in DRC to advise on research activities. The steering committee will be updated yearly on progress and challenges.

## Discussion

The Uvira trial is expected to provide a rigorous, comprehensive assessment of water service infrastructure improvements in terms of diarrhoeal disease and cholera incidence, with the potential to inform implementation, design, and investments in future large-scale water supply interventions for cholera and diarrhoeal disease prevention. This investigation is not only unique due to the nature of the intervention (improvements and extension of piped water supply) but also because of the proposed approach and efforts—including confirmation of suspected cholera cases—to enable rigorous research in a complex setting. Beyond the health impact assessment, by documenting research and intervention processes via multiple sources of information, this study is expected to provide insights into which cholera and diarrhoeal disease prevention mechanisms may be most feasible, economically efficient, and effective. Furthermore, the availability of several years of historical cholera and diarrhoeal disease incidence data provides an opportunity to understand long-term disease transmission trends in an endemic context.

Because this research will be conducted in Eastern DRC, in a challenging context not untypical of cholera transmission hotspots, we have adopted a pragmatic approach to mitigate the impact of pre-identified threats to the protocol. This strategy was adopted in response to the inherent instability of the study setting and complexity of this large-scale infrastructure intervention and a recognition that many potential risks would be beyond the control of the study team or indeed the intervention team. Favourable security, political, administrative, and technical conditions, as well as users’ demand for an improved water service, will be necessary to enable the delivery of the intervention as planned and to execute optimally on all aspects of this study protocol. Here we describe a number of pre-identified risks to the intervention and to the study, and discuss how the design features of our pragmatic strategy can mitigate these.

### Context-specific challenges: security, climate, and administrative processes

Several factors may lead to deviations from the planned timeline for the delivery of the intervention. Given the general volatility and ongoing conflicts in South Kivu [48, 49], construction and research activities could be interrupted at any time by security issues. The condition of existing infrastructures and roads, including poor drainage and heavy rains during the rainy season, represent another potential cause of delays and supply chain disruptions that is beyond the control of the study and intervention teams. In April 2020, extreme flooding affected an estimated 86,000 people in Uvira [59] and damaged the water intake for the water supply network, resulting in an immediate interruption of the water service for a duration that remains undetermined at the time of writing. The strict enforcement of border closures with Burundi and Rwanda due to the COVID-19 pandemic since end of March 2020 further complicated the situation. Ensuring the safety of the construction and research teams is a top priority on this project. Any deviations from the study calendar and scheduled intervention roll-out will be documented carefully by the construction, REGIDESO, and study teams so that they can be accounted for in the analysis. For example, the SW-CRT could be analysed as a non-randomised SW trial if the allocation of cluster randomisation was not or incompletely followed. Additionally, should completion of the works become unfeasible in a given cluster, we will work with the construction team ad hoc to avoid unnecessary delays to the intervention and determine how this study protocol may need to be amended depending on the situation.

### Administrative and technical challenges

The intervention is technically complex and several engineering challenges are expected. In addition, administrative delays relating to, for example, shipping and importation of construction materials, are likely. And, at a management level, the project involves multiple actors, with the national utility, REGIDESO, working in partnership with a third-party contractor, and the VF providing technical support and guidance.

With regard to the protocol, the first challenge lay in obtaining reliable information on the existing infrastructure: inaccurate mapping of the existing water (and other) networks could lead to incomplete and/or incorrect identification of leaks, of pipes requiring rehabilitation, of existing pipes’ depth (for connecting new pipes), and of works planning with respect to other networks. Construction of new, critical infrastructure such as the 2000 m^3^ water tank outside the city also involves coordination at multiple levels to ensure compatibility with electrical installations and land use authorisations. Although hydraulic models will be used to design the intervention, unforeseen impacts of structural modifications and/or increased water demand could affect service quality and require localised adjustments.

Furthermore, one important limitation of the intervention is that it does not address electricity shortages, which are the primary cause of intermittent water supply in Uvira. Early assessments deemed potential solar and hydraulic electricity generation unfeasible or economically not sustainable in the Uvira context. In 2019, REGIDESO reports show that electricity was supplied on average 12 h per day and for less than 6 h on at least 4 days per month. Previous research in Uvira established a clear link between water service interruptions and increases in diarrhoeal disease incidence [60], suggesting that it would be critical to improve service continuity. Infrastructural improvements, including increased pumping and storage capacity, may increase the buffering capacity of the network to deliver water in case of short electricity interruptions; however, these modifications are unlikely to ensure continuity or even reliability (availability of water at known times) of the water supply in case of prolonged power cut. Information collected via cross-sectional household surveys and during the process evaluation will allow us to document and assess water service quality in each cluster throughout the project.

### Economic challenges

From an economic standpoint, the reliance of the intervention on individual demand for new household connections could be a limiting factor, as the requested fee may not be affordable to all Uvira inhabitants. The installation of community taps partially addresses equity concerns, although the water price defined by management committees may still be a barrier to utilisation of these for those living in precarious conditions. Competition between water sales from community taps and individual household connections or use of the latter by multiple households may also affect this impact evaluation. Operational and billing information from REGIDESO will be used in complement to household surveys to better understand water access and compliance with the intervention.

### Research-specific challenges and limitations

From a research standpoint, the fact that the new tank, which is expected to improve service quality in all clusters, will be put into service half-way through cluster-level improvements (Fig. 2) and the flexible step duration (and o verlap between steps) to allow prolonged installation of new household connections—another pragmatic choice required to adapt to the context—both represent deviations from a standard SW-CRT roll-out. Likewise, the heterogeneity in cluster characteristics (Table 1), due to local constraints, is large for a SW-CRT. These aspects will be accounted for in the stepped-wedge analysis by considering potential confounders. An ITS analysis will also be conducted, which does not depend in any way on the intervention structure and implementation.

Spill-over effects could also potentially undermine the study design if inhabitants of a control cluster are able to use an improved “intervention” water service in a neighbouring cluster; for example, if a new private connection is used by multiple households. While household surveys may provide insights into the actual use of water services, we will not be able to systematically assess intervention compliance over the entire city. An intention-to-treat approach will therefore be used in the analysis and a sensitivity analysis will be performed to assess the influence of excluding participants who live within spatial buffers of different widths along the clusters’ borders. Given the predominance of within-household cholera transmission over infection from environmental sources [41, 61], we expect spill-over effects, if any, to remain of limited magnitude.

The reliance on clinical surveillance to assess cholera incidence is another limitation of this evaluation, as it may be influenced by healthcare-seeking behaviour. Although we will not be able to quantify CTC attendance rates for diarrhoeal disease symptoms across Uvira, we will seek to collect information on care habits during the household survey and critically review assumptions related to healthcare use for diarrhoeal diseases. The confirmation of cholera cases by RDTs rather than by culture or molecular methods (PCR) also responds to the need for pragmatic research approaches, as RDTs were identified as the only confirmation method that could be reliably used in the Uvira context considering available resources and laboratory capacity at the time this study started.

The Uvira Health Zone is among those identified as “hotspots” and that will be prioritised for Oral Cholera Vaccination in South Kivu as part of the national strategy to tackle cholera [62]. The deployment of a cholera vaccination campaign—or other cholera and/or COVID-19 control activities—concurrently with water supply improvements that we seek to evaluate could adversely affect our ability to attribute any observed change in cholera incidence to the water supply intervention. Furthermore, since the intervention is expected to simultaneously improve water quality and quantity, our ability to distinguish between the causal effects of these two elements through this trial if a reduction in diarrhoeal disease and/or cholera is observed will remain limited. Considering all the challenges in intervention implementation described above, there is also a possibility that no impact on trial outcomes will be detected despite the efforts deployed by the construction team to follow the study protocol and randomisation. Our hope is that the combination of multiple research and analysis methods in this research project will allow for a comprehensive, well-informed assessment of the water supply intervention and interpretation of its impacts.

## Conclusion

Overall, the Uvira trial is expected to generate new evidence on the impact of large-scale improvements in piped water service in a conflict-affected cholera transmission hotspot. A pragmatic approach is presented in this protocol that should enable conduct of the research in a challenging context without undermining the scientific validity of the trial.

## World Health Organization Trial Registration Data Set

**Table:** 

#	Item	Description
1	Primary Registry and Trial Identifying Number	*clinicaltrials.gov*, NCT02928341
2	Date of Registration in Primary Registry	10th October 2016
3	Secondary Identifying Numbers	LSHTM Ethics Review Board protocols #8913, #10603
4	Source(s) of Monetary or Material Support	French Agency for Development (AFD) Veolia Foundation (VF)
5	Primary Sponsor	London School of Hygiene and Tropical Medicine (LSHTM)
6	Secondary Sponsor(s)	None
7	Contact for Public Queries	Karin Gallandat, Principal Investigator, karin.gallandat@lshtm.ac.uk Keppel St, London, WC1E 7HT, UK + 44 20 76 36 86 36
8	Contact for Scientific Queries	
9	Public Title	Impact Evaluation of Urban Water Supply Improvements on Cholera and Other Diarrhoeal Diseases in Uvira, Democratic Republic of Congo
10	Scientific Title	The impact of improved water supply on cholera and diarrhoeal diseases in Uvira, Democratic Republic of the Congo: a pragmatic stepped-wedge cluster randomised trial and economic evaluation
11	Country of Recruitment	Democratic Republic of the Congo
12	Health Condition(s) or Problem(s) Studied	Cholera, diarrhoeal disease
13	Intervention	Control: current water supply service Intervention: improved water supply service (new and rehabilitated supply network infrastructure including community and household tap connections)
14	Key Inclusion and Exclusion Criteria	Inclusion Criteria: Uvira resident (without restriction placed on age or sex) admitted to the Uvira Cholera Treatment Centre at the General Hospital in Uvira Exclusion Criteria: Any patients admitted during the period of intervention implementation in each cluster
15	Study Type	Interventional; stepped-wedge cluster randomised trial
16	Date of First Enrolment	10th October 2016
17	Sample Size	Planned enrolment: 5000 Enrolment to date: 4280
18	Recruitment Status	Recruiting
19	Primary Outcome(s)	(1) monthly incidence of suspected cholera cases, as measured by the number of CTC admissions attributed to each cluster; (2) monthly incidence of confirmed cholera cases, based on rapid detection test (RDT) results for consenting patients
20	Key Secondary Outcomes	None
21	Ethics Review	Approved. LSHTM Ethics Committee: #8913, 14th December 2015; #10603, 7th April 2016 Kinshasa School of Public Health Ethics Committee: ESP/CE/088/2015, 4th September 2015 (last renewal: ESP/CE/143/2020, 23rd August 2020)
22	Completion Date	Expected 27th September 2021
23	Summary Results	Not available
24	IPD Sharing Statement	No IPD sharing

## Supplementary Information

**Additional file 1.** SPIRIT check-list.

**Additional file 2.** Consent form.

## Data Availability

Any use of trial data will be controlled by the Principal Investigators. All final, anonymised datasets and statistical analysis codes will be made available on a public repository (e.g. Open Science Framework, osf.io) at the time of publication of trial results.

## Abbreviations

AFD: Agence Française de Développement
CBA: Cost-benefit analysis
CEA: Cost-effectiveness analysis
CONSORT: Consolidated Standards of Reporting Trials
CTC: Cholera Treatment Centre
DALY: Disability-adjusted life years
DRC: Democratic Republic of the Congo
ETEC: Enterotoxigenic *Escherichia coli*
GEE: Generalised estimating equations
GPS: Global Positioning System
GTFCC: Global Task Force on Cholera Control
ICC: Intraclass correlation coefficient
ITS: Interrupted time series
LSHTM: London School of Hygiene and Tropical Medicine
MDD: Minimum detectable difference
NGO: Non-governmental organisation
OCV: Oral cholera vaccine
RDT: Rapid detection test
SDG: Sustainable Development Goals
SW-CRT: Stepped-Wedge Cluster Randomised Trial
VF: Veolia Foundation
WASH: Water, sanitation and hygiene
WHO: World Health Organization
