# Supplementary material for: The impact of improved water supply on cholera and diarrhoeal diseases in Uvira, Democratic Republic of the Congo: a protocol for a pragmatic stepped-wedge cluster randomised trial and economic evaluation
Source: Trials. 2021 Jun 21;22:408. doi: 10.1186/s13063-021-05249-x (PMC8215491; doi:10.1186/s13063-021-05249-x)
Supplement: Supplementary file 2 — Additional file 2. Consent form. [file 13063_2021_5249_MOESM2_ESM.pdf]

PARTICIPANT INFORMATION SHEET

Study title:

Cholera confirmation amongst patients admitted to the cholera treatment centre in Uvira, DR Congo

You are being invited to take part in a research study led by the London School of Hygiene and Tropical Medicine in the United Kingdom in collaboration with the Uvira Health Zone. Before you decide whether to participate, it is important for you to understand why the research is being done and what it will involve. Please ask us if there is anything that is not clear or if you would like more information, and please take time to decide whether or not you wish to take part. The following is to explain the details of the study:

1. What is the purpose of the study?

Water access is key to prevent diarrhoeal disease and cholera, which has been affecting the Uvira population for many years. Cholera is a disease caused by the cholera bacteria and characterised by acute watery diarrhoea. Symptoms similar to cholera can be caused by other diarrhoeal disease agents (microbes) and lead someone to seek care at the cholera treatment centre.

We are conducting an evaluation to understand whether improvements of the REGIDESO water supply network can reduce the number of diarrhoeal disease and cholera cases. We therefore would like to understand how many of the patients treated at the cholera treatment centre are confirmed cholera cases (infected with the cholera bacteria).

2. Why have I been chosen?

You are being asked to participate in this study because you or your child sought care at the cholera treatment centre of the Hôpital Général in Uvira. We are inviting all patients from the cholera treatment centre to participate.

3. Do I have to take part?

It is up to you to decide whether to join the study. We will describe the study and go through this information sheet and, if you agree to take part, we will then ask you to sign a consent form. You are free to withdraw from the study at any time, without giving a reason. If you decide to withdraw, with your permission, we will record your reason for withdrawing. Your decision to join the study or not will not affect the health services that are available to you or your child.

4. What will happen to me if I take part?

If you agree to participate in the study, a trained nurse will take a stool sample by rectal swab from you or your child. She will explain the procedure to you before doing it.

5. What are the possible disadvantages and risks of taking part?

Collecting the stool sample will cause no more than minimal discomfort and does not pose a risk to your or your child's health. The procedure will be overseen by trained medical staff.

6. What are the possible benefits of taking part?

Participation in the study will not help you or your child directly but the information we collect may lead to improvements in how cholera is managed in the region and support more effective prevention strategies.

7. Will my taking part in the study be kept confidential?

Yes. All information collected about you or your child, including analysis results, will be kept strictly confidential and anonymous.

8. What will happen if I don't want to carry on with the study?
You can decide to stop participating in the study at any time, even after you gave consent and you or your child provided a sample. If you withdraw from the study, you can decide whether you want us to destroy the sample or whether you allow us to test it.

9. Who will pay the costs that I may incur through participating in this study?
Your participation in the study is not expected to generate any expenses for you.

10. What if something goes wrong?
If you have a concern about any aspect of this study, you should ask to speak to the local study supervisor, who will try to answer your questions: **Jaime Mufitini Saidi (T. 099 2 023 652).**

If you wish to complain formally, or have any concerns about any aspect of the way you have been treated, you should immediately inform the Principal Investigator: Dr **Karin Gallandat (karin.gallandat@lshtm.ac.uk, +44 20 7636 8636).**

The London School of Hygiene & Tropical Medicine holds insurance policies which apply to this study. If you experience harm or injury as a result of taking part in this study, you may be eligible to claim compensation.

12. What will happen to the samples and the results of the research study?
Stool samples (rectal swabs) will be collected at the cholera treatment centre and put in a liquid that allows cholera bacteria to grow. After a few hours, the laboratory technician from the cholera treatment centre will test whether this liquid contains cholera bacteria. If so, a few drops of the liquid will be placed on filter paper and shipped to Institut Pasteur, in Paris (France), for further analyses that will help understand cholera transmission dynamics in the region. All the samples testing results will be analysed by researchers working with the London School of Hygiene and Tropical Medicine. Overall results of the study will be summarized anonymously and will only be presented in aggregated form, without any identifying information. It is expected that study results will be published and shared after completion of the study with partner organizations, including the Ministry of Health in DR Congo.

13. Where can you find out more about how your information is used?
You can find out more about how your information and information about your child will be used:
- At https://www.lshtm.ac.uk/files/research-participant-privacy-notice.pdf
- by asking one of the research team
- by sending an email to DPO@lshtm.ac.uk

You will be given a copy of the information sheet and a signed consent form to keep.

Thank you for considering taking the time to read this sheet.

INFORMED CONSENT FORM

Cholera confirmation amongst patients admitted to the cholera treatment centre in Uvira, DR Congo

Principal Investigator: Oliver Cumming (LSHTM)

	**Please initial each box**
1. I confirm that I have read and understand the participant information sheet dated XX.XX.XXXX (Version 1) for the above study. I have had the opportunity to consider the information, ask questions and have had these answered fully.	
2. I understand that my participation is voluntary and I am free to withdraw at any time, without giving any reason, without my medical care or legal rights being affected.	
3. I understand that data collected during the study, including results from samples analyses, may be looked at by responsible individuals from the London School of Hygiene & Tropical Medicine where it is relevant to their participation in this research. I give permission for these individuals to access that information.	
4. I agree to take part in the above study.	

Name of Participant *(printed)*	Signature/Thumbprint	Date (dd/mm/yyyy)
Name of Person taking consent (*printed*)	Signature	Date (dd/mm/yyyy)

If the participant is unable to sign (otherwise enter "N/A" in fields): As a witness, I confirm that all the information about the study was given and the participant consented to taking part.

Name of Impartial Witness (*printed*)	Signature	Date (dd/mm/yyyy)

1 copy for participant; 1 copy for study team to be retained
